# Combination of EGFR-TKI and Chemotherapy Versus EGFR-TKI Monotherapy as Neoadjuvant Treatment of Stage III-N2 EGFR-Mutant Non-Small Cell Lung Cancer

**DOI:** 10.1093/oncolo/oyae052

**Published:** 2024-03-25

**Authors:** Yingqi Xu, Hao Ji, Yidan Zhang, Liwen Xiong, Baohui Han, Hua Zhong, Jianlin Xu, Runbo Zhong

**Affiliations:** Department of Respiratory and Critical Care Medicine, Shanghai Chest Hospital, Shanghai Jiao Tong University School of Medicine, Shanghai, People’s Republic of China; Department of Respiratory and Critical Care Medicine, Department of Healthcare-Associated Infection Management, Shanghai Chest Hospital, Shanghai Jiao Tong University School of Medicine, Shanghai, People’s Republic of China; Department of Respiratory and Critical Care Medicine, Shanghai Chest Hospital, Shanghai Jiao Tong University School of Medicine, Shanghai, People’s Republic of China; Department of Respiratory and Critical Care Medicine, Shanghai Chest Hospital, Shanghai Jiao Tong University School of Medicine, Shanghai, People’s Republic of China; Department of Respiratory and Critical Care Medicine, Shanghai Chest Hospital, Shanghai Jiao Tong University School of Medicine, Shanghai, People’s Republic of China; Department of Respiratory and Critical Care Medicine, Shanghai Chest Hospital, Shanghai Jiao Tong University School of Medicine, Shanghai, People’s Republic of China; Department of Respiratory and Critical Care Medicine, Shanghai Chest Hospital, Shanghai Jiao Tong University School of Medicine, Shanghai, People’s Republic of China; Department of Respiratory and Critical Care Medicine, Shanghai Chest Hospital, Shanghai Jiao Tong University School of Medicine, Shanghai, People’s Republic of China

**Keywords:** NSCLC, EGFR, TKI, neoadjuvant treatment, chemotherapy

## Abstract

**Background:**

The efficacy of neoadjuvant treatment with epidermal growth factor receptor tyrosine kinase inhibitor (EGFR-TKI) monotherapy in patients with stage III-N2 EGFR-mutant remains unsatisfactory. This study explored the potential benefits of combining first-generation EGFR-TKI with chemotherapy as a neoadjuvant treatment for patients with stage III-N2 EGFR-mutant non-small cell lung cancer (NSCLC).

**Patients and Methods:**

The medical records of patients with III-N2 EGFR-mutant NSCLC who received neoadjuvant therapy with EGFR-TKI at Shanghai Chest Hospital from October 2011 to October 2022 were retrospectively reviewed. Patients with stage III-N2 EGFR-mutant NSCLC who received first-generation TKI combined with chemotherapy as neoadjuvant treatment were included in the combination group, and those who received EGFR-TKI monotherapy were included in the monotherapy group. The study assessed the objective response rate (ORR) according to Response Evaluation Criteria in Solid Tumors (RECIST) version 1.1, disease-free survival (DFS), overall survival (OS), downstaging rates of pathologic lymph nodes (from stage N2 to N1 or N0), major pathologic response (MPR) rate, pathological complete response (PCR) rate, and safety.

**Results:**

A total of 74 631 patients with EGFR-mutant NSCLC were screened, and 60 patients were included, 7 of whom did not undergo surgery after neoadjuvant targeted therapy. Of the remaining 53 patients, 15 received first-generation EGFR-TKI combined with chemotherapy as neoadjuvant treatment, and 38 received EGFR-TKI monotherapy. The median follow-up time was 44.12 months. The ORR was 50.0% (9/18) in the combination group and 40.5% (17/42) in the monotherapy group (*P* = .495). The MPR rate was 20.0% (3/15) and 10.5% (4/38) in the combination and monotherapy groups, respectively (*P* = .359). No patients achieved PCR in the combination group, while 3 (7.89%) attained PCR in the monotherapy group. The 2 groups did not differ in N2 downstaging rate (*P* = .459). The median DFS was not reached in the combination group, while it was 23.6 months (95% CI: 8.16-39.02) in the monotherapy group (*P* = .832). Adverse events observed were consistent with those commonly associated with the 2 treatments.

**Conclusion:**

Combination therapy with first-generation EGFR-TKI and chemotherapy could be considered a neoadjuvant treatment option for patients with stage III-N2 EGFR-mutant NSCLC, exhibiting acceptable toxicity. However, regarding short-term efficacy, combination therapy did not demonstrate superiority over EGFR-TKI monotherapy. Long-term follow-up is warranted for a more accurate assessment of the DFS and OS.

## Teaser

This study explored the potential benefits of combining first-generation epidermal growth factor receptor (EGFR)-tyrosine kinase inhibitor with chemotherapy as a neoadjuvant treatment for patients with stage III N2 EGFR-mutant non-small cell lung cancer.

**Implications for Practice**

This study explores the possible benefit of chemotherapy combined with the first-generation tyrosine kinase inhibitor (TKI) as neoadjuvant therapy for epidermal growth factor receptor (EGFR)-mutated III-N2 NSCLC. We found that chemotherapy combined with first-generation EGFR-TKI could be used as neoadjuvant therapy for EGFR-mutated III-N2 NSCLC with acceptable toxicity. Besides, PCR was found in 7.89% (*n* = 3) of patients in the monotherapy group, implying that even first-generation EGFR-TKIs may still have a good therapeutic effect in stage III patients with EGFR mutations as neoadjuvant therapy.

## Introduction

It is well-established that stage III non-small cell lung cancer (NSCLC) is characterized by significant heterogeneity. While patients with stage IIIC and the majority of stage IIIB are classified as unresectable, maintenance therapy with durvalumab after concurrent chemoradiation has become the standard of care for these patients, resulting in a 5-year survival rate of 42.9%.^1^ Patients with NSCLC with stage IIIA and a proportion of stage IIIB are classified as resectable or potentially resectable and may benefit from surgery. Neoadjuvant chemotherapy can improve the 5-year overall survival (OS) by approximately 5% (from 20% to 25%)^2^ in patients with stage IIIA. The emergence of neoadjuvant immunotherapy^3^ has significantly improved the event-free survival (EFS) and pathological complete response (PCR) in patients with driver gene-negative profiles ranging from stage IB to IIIA. In recent years, neoadjuvant immunotherapy has become one of the preferred treatment options for patients with driver-negative stage IIIA. However, neoadjuvant therapy for patients who harbor epidermal growth factor receptor (EGFR), especially for those in stage III-N2 NSCLC, remains to be further optimized.

EGFR mutations have been documented in 11% of Caucasian and 50% of Asian populations with lung adenocarcinoma.^4^ EGFR-tyrosine kinase inhibitors (TKI) are the standard first-line treatment and have shown a significant survival benefit for patients with advanced NSCLC with EGFR-sensitive mutations.^5,6^ In the neoadjuvant setting, EGFR-TKIs have also shown therapeutic advantages in patients with IIIA-N2 with EGFR mutations. Several studies have evaluated the effect of first-generation EGFR-TKI as neoadjuvant therapy in patients with stage IIIA-N2 with EGFR mutations.^7-10^ These studies reported improvements in disease-free survival (DFS) in patients with IIIA-N2 EGFR-mutant treated with neoadjuvant first-generation EGFR-TKIs compared with chemotherapy. Nevertheless, the improvement in objective response rate (ORR) remains limited, and there has been no significant improvement in OS. Thus, there is an ongoing need for improved therapeutic approaches for this patient population.

In the NEJ009 study, the combination of chemotherapy and first-generation EGFR-TKI achieved a better ORR (84%) compared with EGFR-TKI monotherapy (67%) in advanced NSCLC with EGFR mutations, as well as a significant improvement in PFS.^11^ Although there was no significant difference in OS between the 2 groups, the combination of chemotherapy and first-generation EGFR-TKI demonstrated better short-term efficacy compared to EGFR-TKI monotherapy. This suggests that combination therapy may significantly increase the ORR and major pathological response (MPR) in patients with stage III-N2 NSCLC with EGFR mutations when used as neoadjuvant treatment. Since OS has been found to be significantly associated with MPR,^12,13^ it would be worthwhile to investigate whether the combination of chemotherapy with EGFR-TKI as neoadjuvant treatment can improve the OS in patients with NSCLC with EGFR mutations and stage III-N2 disease. This study aims to determine the potential benefits of chemotherapy combined with first-generation EGFR-TKIs as neoadjuvant in patients with stage III-N2 NSCLC with EGFR mutation.

## Methods

### Patients

**Figure 1.**
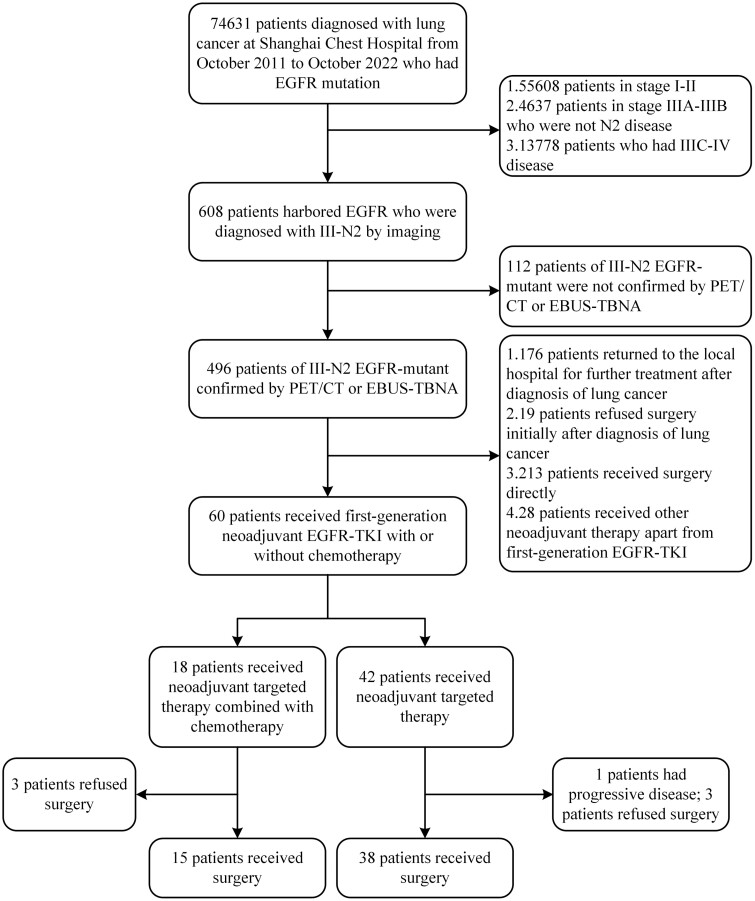
CONSORT diagram.

In this study, we reviewed the medical history of 74 631 patients with lung cancer with EGFR mutations at Shanghai Chest Hospital from October 2011 to October 2022. The inclusion criteria consisted of (1) patients with histologically confirmed NSCLC who harbored sensitive EGFR mutations, specifically EGFR exon 19 deletion or exon 21 L858R mutation confirmed by ARMS or NGS; (2) patients diagnosed with clinical stage III-N2 (IIIA-N2 and IIIB [T3-4 N2]) based on imaging studies combined with endobronchial ultrasound-guided transbronchial needle aspiration (EBUS-TBNA) or positron emission tomography/computed tomography (PET/CT) at initial diagnosis, according to the eighth edition of the tumor/node/metastasis (TNM) staging system; (3) patients treated with a combination of chemotherapy and first-generation EGFR-TKI or first-generation EGFR-TKI monotherapy as neoadjuvant treatment. The exclusion criteria included patients with other malignancies, concurrent small cell carcinoma, those who received alternative treatments in the neoadjuvant phase, or those without complete survival information (Fig. 1).

This retrospective study was approved by the Ethics Committee of Shanghai Chest Hospital (ethical approval number: IS23024) and was performed in accordance with the principles of the Helsinki Declaration of 1964 (revised in 2013). The need for informed consent from the patients was waived.

### Clinical Assessments and Follow-Up

Staging assessments were conducted in all patients at initial diagnosis, at the end of neoadjuvant therapy, and after surgery according to the eighth edition of the TNM classification system. During the neoadjuvant treatment period, treatment efficacy was assessed by a chest CT scan performed at intervals of 1-2 months until termination of neoadjuvant treatment.

The key assessment indicators included DFS, OS, pathological downstaging rates of lymph nodes (from stage N2 to stage N1 or stage N0), ORR, MPR, PCR, and adverse events (AEs).

The best objective responses (BORs) were evaluated according to Response Evaluation Criteria in Solid Tumors (RECIST v1.1), including complete response (CR), partial response (PR), stable disease (SD), or progressive disease (PD). ORR was defined as the proportion of patients achieving CR or PR radiologically. The disease control rate (DCR) was defined as the proportion of patients who achieved CR, PR, and SD based on imaging assessments. The DFS was calculated from the end of surgery to disease progression, death, or the last follow-up visit, whichever came first. The OS was calculated from the beginning of treatment to death, or the last follow-up visit, whichever came first. MPR was defined as less than 10% residual viable tumor (RVT) cells, while PCR was defined when no residual viable tumor cells were detected in the primary tumor and sampled lymph nodes pathologically. AEs were categorized according to the Medical Dictionary for Regulatory Activities (MedDRA v20.0) and graded according to the Common Terminology Criteria for Adverse Events (CTCAE v5.0).

Patients underwent long-term follow-up after surgery, including a chest CT scan every 3-6 months, and abdominal ultrasound, cranial magnetic resonance imaging, and bone emission computed tomography if necessary. The last follow-up date for the study was March 24, 2023.

### Statistical Analysis

Statistical analyses were performed using SPSS 20.0 statistical software (IBM, Armonk, NY, USA). The age of patients was presented as the median and range, while other patient characteristics were presented as categorical variables, indicating the number and percentage of patients. BORs and MPR were compared using the chi-square test. Survival outcomes (DFS and OS) were estimated by the Kaplan-Meier method and compared using the log-rank test. Multivariate Cox regression was used to identify significant factors related to DFS and OS. Data were presented as median values with their respective 95% CIs. A *P*-value ≤ .05 was statistically significant.

## Results

### Baseline Characteristics

A total of 74 631 patients with EGFR-mutated NSCLC who attended Shanghai Chest Hospital from October 2011 to October 2022 were screened. Among them, 608 patients were diagnosed with stage III-N2 by imaging at initial diagnosis. After further evaluation, 496 patients were confirmed to have N2 disease by enhanced CT combined with PET/CT or EBUS-TBNA. Sixty patients received first-generation EGFR-TKI combined chemotherapy or EGFR-TKI monotherapy as neoadjuvant treatment, and all patients received at least 2 treatment courses. Subsequently, 3 patients in each group (combination and monotherapy) declined surgery, while one patient in the EGFR-TKI monotherapy group did not undergo surgery due to disease progression. A total of 53 patients underwent surgery, 15 in the combination group and 38 in the monotherapy group (Fig. 1).

The patient population consisted predominantly of females (56.7%) and never smokers (70.0%), and the most common histological type was adenocarcinoma (95.0%). Among the observed EGFR mutations, 56.6% were identified as EGFR exon 19 deletion, while 43.4% were identified as EGFR exon 21 mutation. At the time of initial diagnosis, the majority of patients in both the combination group (61.1%) and the monotherapy group (47.5%) were classified as clinical stage T2N2. The diagnosis of N2 disease was determined by PET in 55.6% of patients in the combination group and 45.0% in the monotherapy group, while EBUS-TBNA confirmed N2 disease in 44.4% of patients in the combination group and 55.0% in the monotherapy group before neoadjuvant treatment. The median treatment duration for patients receiving combination therapy was 3 months. The patients in the combination group received pemetrexed 500 mg/m^2^ and carboplatin AUC 5.0 every 3-4 weeks with oral targeted therapy concomitantly. The median treatment duration for patients receiving targeted monotherapy neoadjuvant treatment was 2 months. The targeted therapeutic drugs received by the 2 groups are shown in Supplementary Table S1. The duration of treatment received before surgery is shown in Supplementary Table S2.

Demographic and baseline characteristics of patients who underwent surgery are shown in Table 1.

**Table 1.** Characteristics of patients who received EGFR-TKI combined with chemotherapy or monotherapy as neoadjuvant treatment.

Characteristics	EGFR-TKI (*n* = 38) (%)	EGFR-TKI combined with chemotherapy (*n* = 15) (%)	*P* value
Sex			.459
Male	16 (42.1)	8 (53.3)	
Female	22 (57.9)	7 (46.7)	
Median age, years (range)	63 (38-82)	57 (44-73)	.034
Smoking status			.754
Non-smokers	27 (71.1)	10 (66.7)	
Smokers	11 (28.9)	5 (33.3)	
ECOG PS score			.131
0	2 (5.3)	3 (20.0)	
1	36 (94.7)	12 (80.0)	
Pathological type			>.99
Adenocarcinoma	36 (94.7)	14 (93.3)	
Nonadenocarcinoma	2 (5.3)	1 (6.7)	
Method of preoperative diagnosis			—
CT-guided percutaneous lung puncture	13 (34.2)	3 (20.0)	
EBUS-TBNA	16 (42.1)	4 (26.7)	
TBB/TBLB	9 (23.7)	8 (53.3)	
T stage (before surgery)			—
T1a	0 (0.0)	0 (0.0)	
T1b	1 (2.6)	1 (6.7)	
T1c	9 (23.7)	3 (20.0)	
T2a	11 (28.9)	3 (20.0)	
T2b	6 (15.8)	6 (40.0)	
T3	8 (21.1)	2 (13.3)	
T4	3 (7.9)	0 (0.0)	
T stage (after surgery)			—
T1a	4 (10.5)	3 (20.0)	
T1b	4 (10.5)	2 (13.3)	
T1c	7 (18.4)	5 (33.3)	
T2a	8 (21.1)	1 (6.7)	
T2b	15 (39.5)	3 (20.0)	
T3	0 (0.0)	1 (6.7)	
T4	0 (0.0)	0 (0.0)	
Method of N2 preoperative diagnosis			—
EBUS-TBNA	22 (57.9)	7 (46.7)	
PET/CT	16 (42.1)	8 (53.3)	
N2 status (before surgery)			.437
Single-station N2	11 (28.9)	6 (40.0)	
Multi-station N2	27 (71.1)	9 (60.0)	
N status (after surgery)			—
N0	14 (36.8)	8 (53.3)	
N1	2 (5.3)	0 (0.0)	
N2	22 (57.9)	7 (46.7)	
EGFR-activating mutations			.006
EGFR exon 19 deletion	26 (68.4)	4 (26.7)	
EGFR exon 21 mutation	12 (31.6)	11 (73.3)	

**Figure 2.**
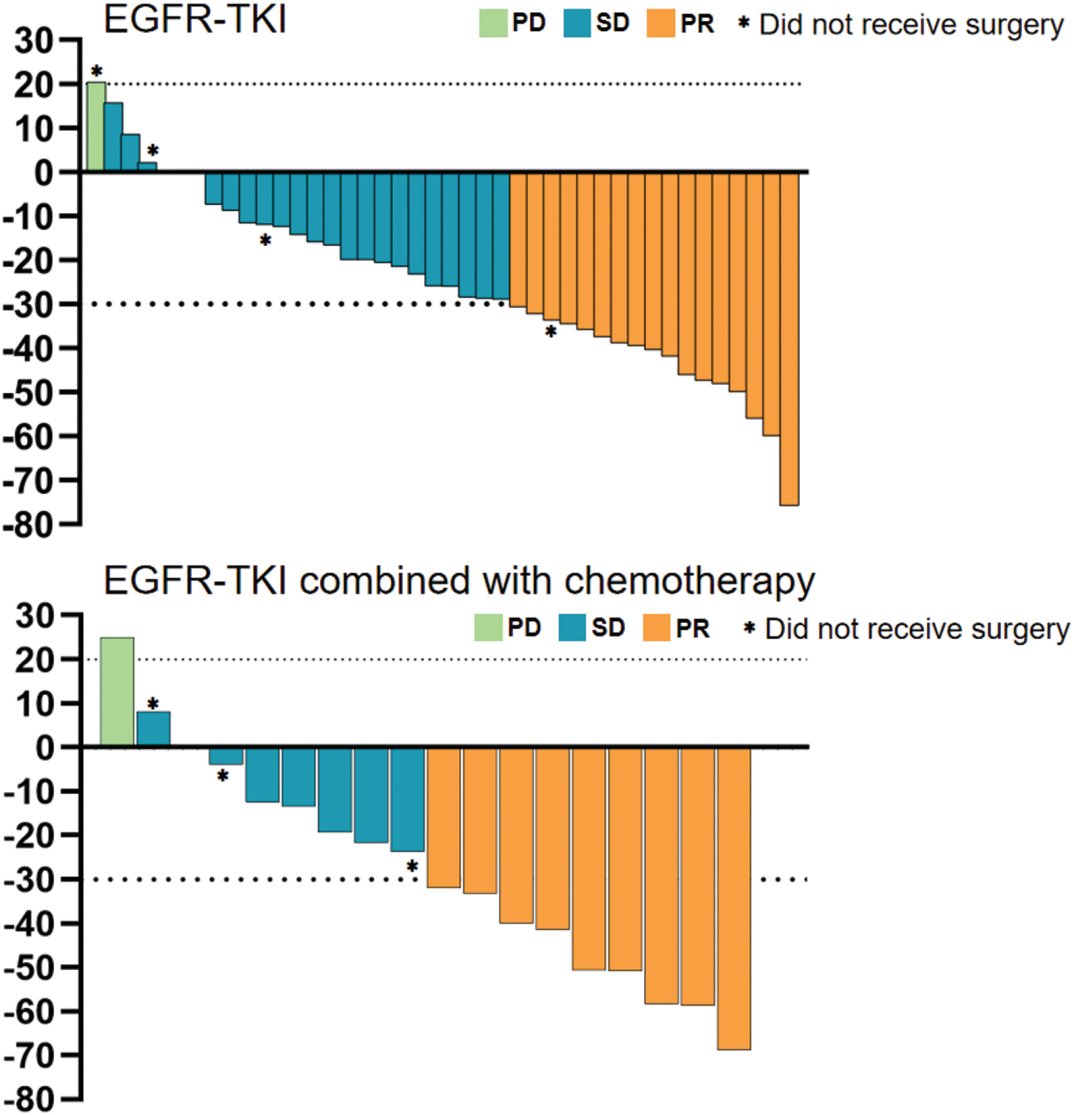
Waterfall plot of optimal preoperative changes in target lesion from baseline for patients who received neoadjuvant EGFR-TKI therapy or combined therapy.

The median follow-up time in our study was 44.12 months. The median time intervals from the end of treatment to radiographic assessment and surgical resection were 0 (−19 to 10) days and 8 (4-36) days, respectively. In the combination group, out of the 15 patients, 9 patients achieved PR, 5 patients achieved SD, and 1 patient achieved PD. The patients with PD underwent R1 resection, while the remaining underwent R0 resection. Among the 38 patients in the monotherapy group, 16 patients achieved PR, 22 patients achieved SD. One patient with SD underwent R1 resection, and the rest underwent R0 resection. The ORR was 50.0% (9/18) in the combination group and 40.5% (17/42) in the monotherapy group, respectively (*P* = .495). The DCR rate was 94.4% (17/18) in the combination group and 97.6% (41/42) in the monotherapy group (*P* = .530). A waterfall plot of optimal preoperative changes in target lesions from baseline for patients receiving neoadjuvant therapy is shown in Fig. 2.

No patients in the combination group achieved CR radiologically (0/15, 0%) or pathologically (0/15, 0%). In the monotherapy group, no patients achieved CR radiologically (0/38, 0%), and 3 patients achieved CR pathologically (3/38, 7.89%). The *P*-value for comparing the PCR between the 2 groups was 0.263. The incidence rates of MPR in the combination and monotherapy groups were 20.0% (3/15) and 10.5% (4/38), respectively (*P* = .359; Table 2). Only 1 patient in the monotherapy group who achieved PCR developed pleural metastasis 6 months after surgery, and the remaining patients in both groups who achieved PCR or MPR did not relapse or die at the last follow-up. The rate of pathological downgrading from N2 to N1/N0 was 53.3% (8/15) in the combination group and 42.1% (16/38) in the monotherapy group, showing no statistically significant difference (*P* = .459).

**Table 2.** The proportion of residual viable tumor cells after neoadjuvant treatment.

	EGFR-TKI (*n* = 38) (%)	EGFR-TKI combined with chemotherapy (*n* = 15) (%)
0%	3(7.9)	0(0.0)
0%<*x* ≤ 10%	1(2.6)	3(20.0)
10%<*x* ≤ 50%	3(7.9)	3(20.0)
≥50%	31(81.6)	9(60.0)

**Figure 3.**
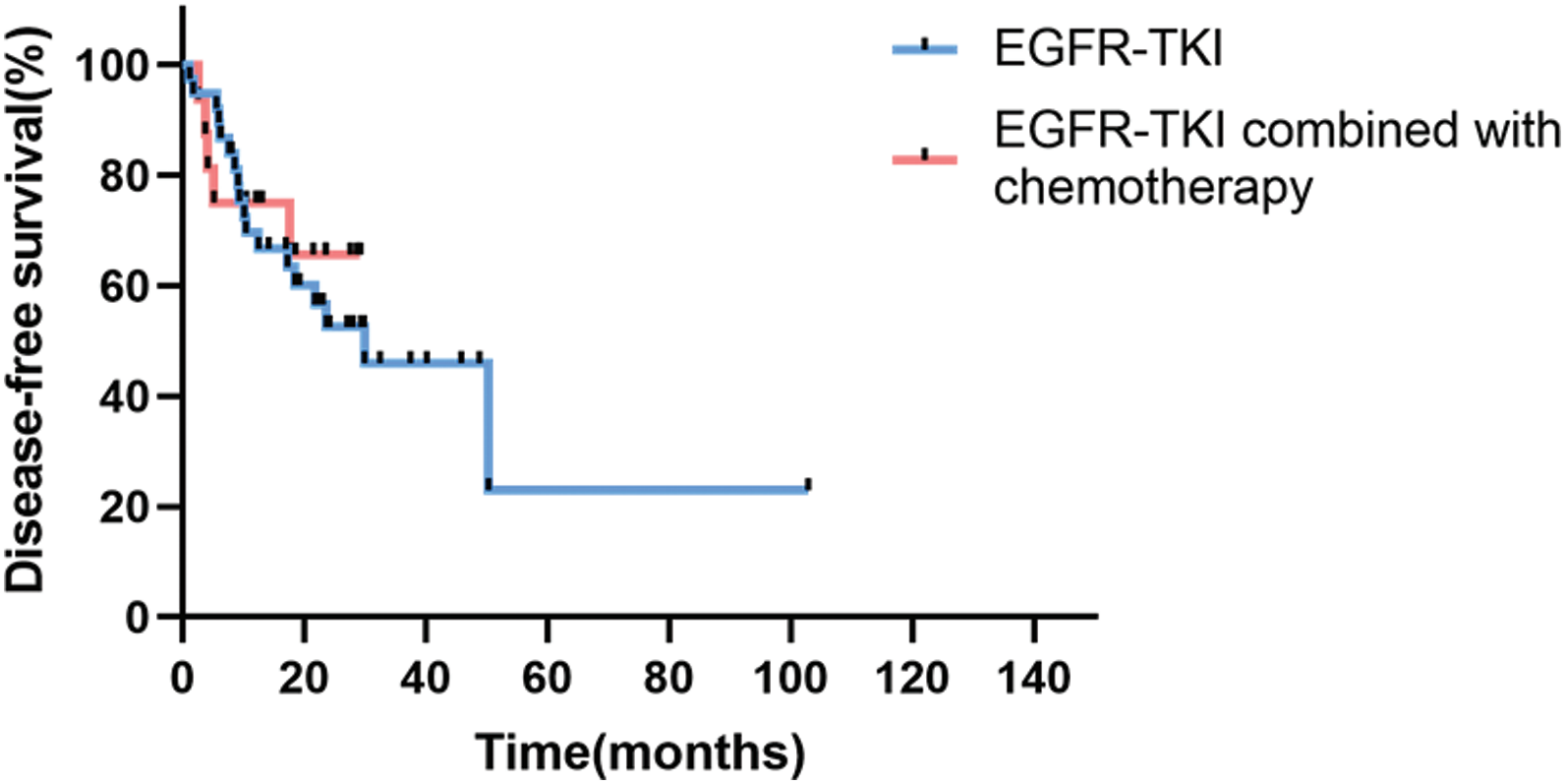
Kaplan-Meier analysis of DFS.

**Figure 4.**
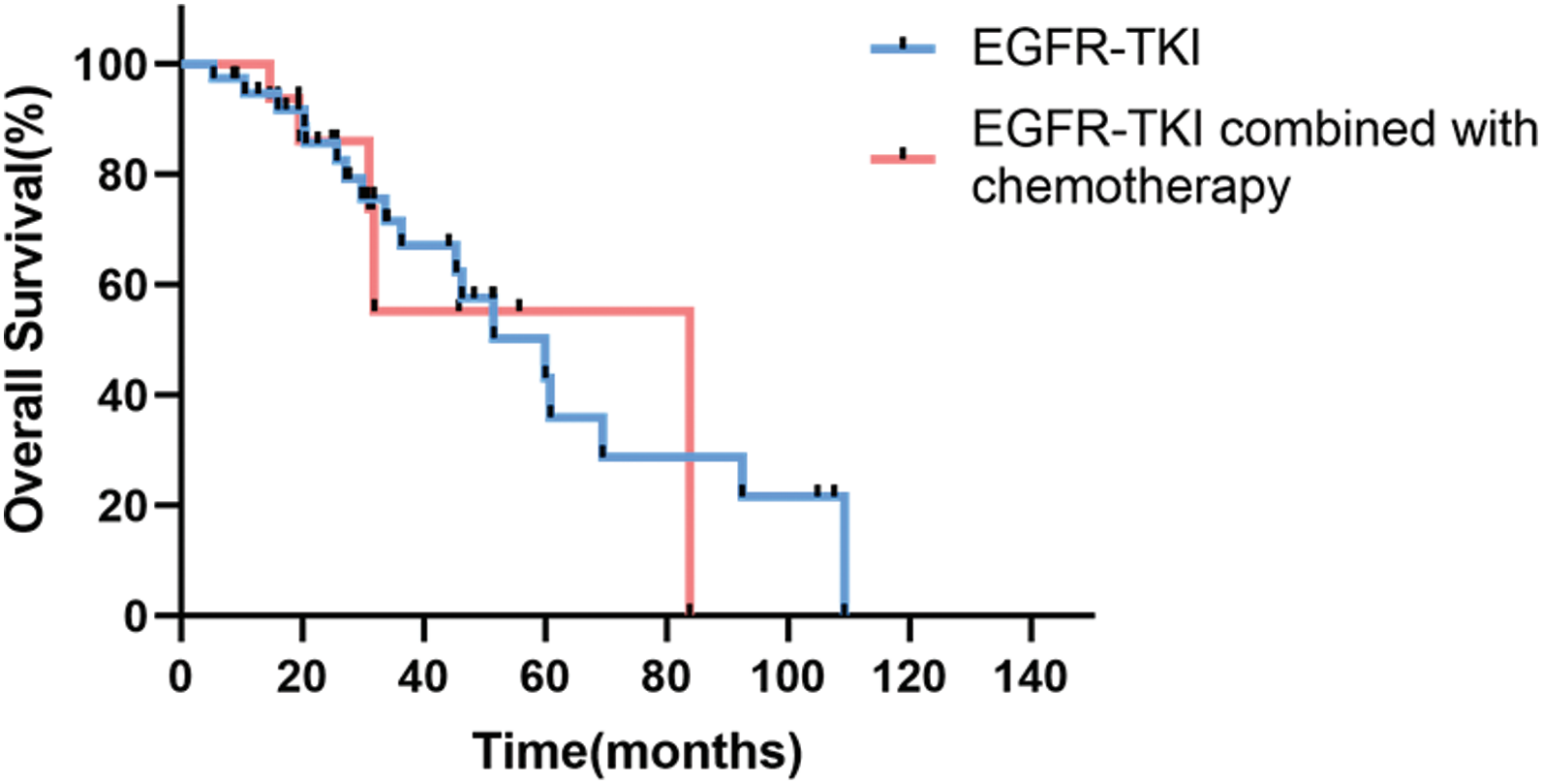
Kaplan-Meier analysis of OS.

At the last follow-up, the recurrence rate was 40.0% (6/15) in the combination group and 52.6% (20/38) in the monotherapy group (*P* = .407). Twenty-two deaths were reported, including 4 in the combination group and 18 in the monotherapy group. The median DFS in the combination group could not be determined, while it was 23.6 months in the monotherapy group (95 CI%, 8.16-39.02; *P* = .832; Fig. 3). The DFS rate at 12 months in the combination group was 60.0%, while it was 63.2% in the monotherapy group (*P* = .481). The median OS was 83.81 months (95 CI%, not available) in the combination group versus 60.02 months (95 CI%, 38.54-81.50) in the monotherapy group (*P* = .809; Fig. 4). Neither DFS nor OS showed a statistically significant difference between the 2 groups, although the follow-up was relatively short (median follow-up time, 44.12 months).

**Figure 5.**
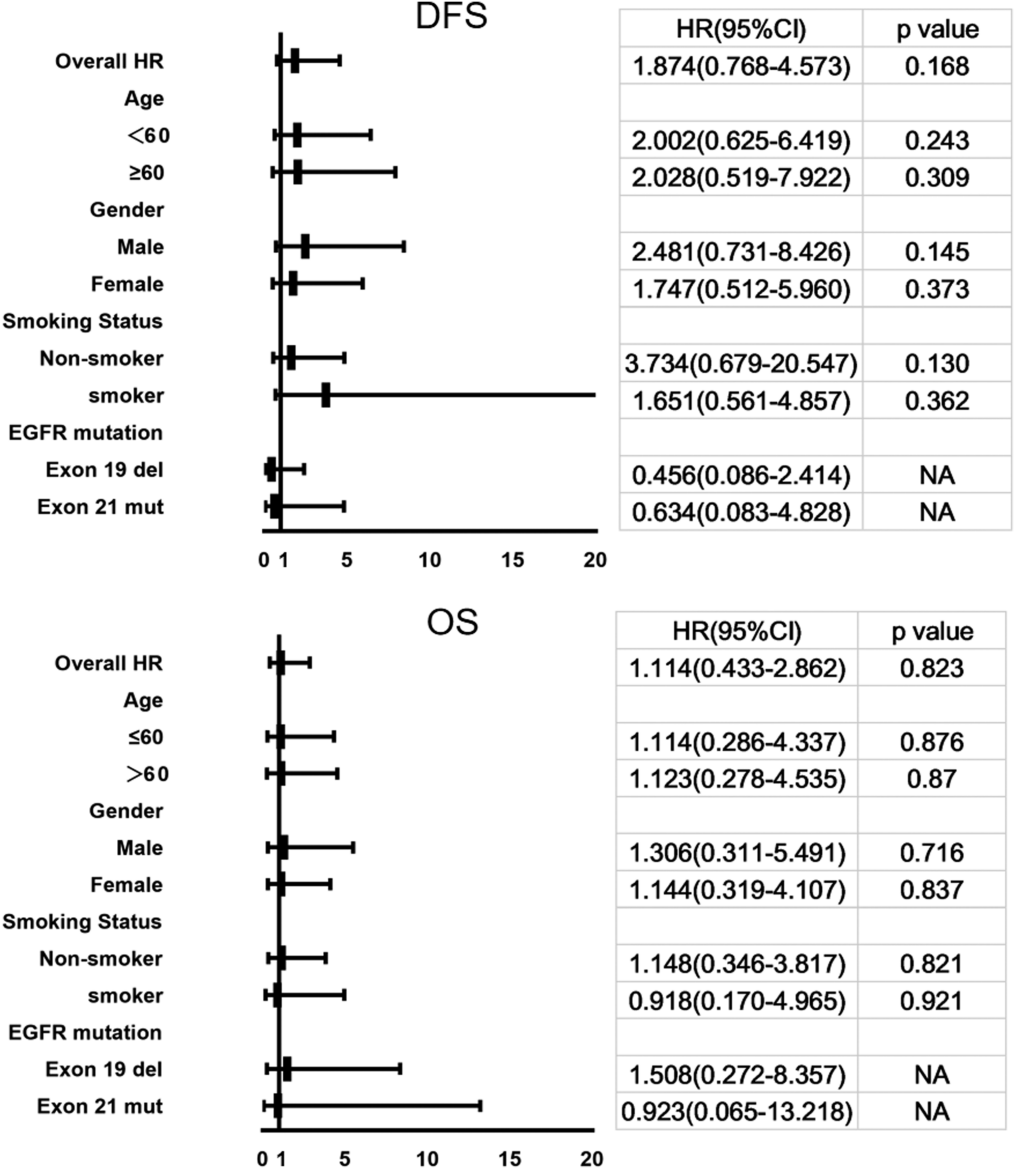
Multivariate Cox regression related to DFS and OS.

Subgroup analyses of DFS and OS did not show significant differences between the 2 groups in age (<60 and ≥60 years), sex (male and female), smoking status (nonsmoker and smoker), and EGFR mutation type (exon 19 deletion and exon 21 L858R mutation). The forest plot for subgroup analysis is shown in Fig. 5.

Twelve (75%) patients in the combination group and 27 (71.1%) patients in the monotherapy group received postoperative adjuvant therapy, respectively (Supplementary Table S3). Targeted therapy was the most frequently used adjuvant treatment after surgery, administered in 40.0% (6/15) of patients in the combination group and 28.9% (11/38) of patients in the monotherapy group, respectively (Supplementary Table S4).

No significant differences in DFS were identified between patients who received TKI as adjuvant therapy and those who received treatments other than TKI in monotherapy and combination groups, which may be attributed to the limited sample size and variations in the duration of EGFR-TKI treatment.

### Safety

In our study, no patients discontinued monotherapy or combination therapy due to AEs. Supplementary Table S5 provides details on AEs associated with neoadjuvant therapy. Six (10.0%) patients experienced grade 3 or greater toxicities during treatment with neoadjuvant therapy. Anorexia (42.9%), rash (38.1%), diarrhea (26.2%), and abnormal liver function (16.6%) were the most common AEs in the EGFR-TKI monotherapy arm. Anorexia (66.6%), nausea (61.0%), fatigue (44.4%), rash (38.8%), and decreased WBC count (38.8%) were most common in the combined therapy group. Grade 3/4 toxicities occurred less frequently in the monotherapy group (7.1%) than in the combined group (16.6%). AEs were all manageable and reversible. No grade 4 or 5 adverse events were reported.

## Discussion

Previous studies^8,14,15^ have focused on the role of EGFR-TKI in the neoadjuvant setting for EGFR-mutated III-N2 NSCLC. Nevertheless, the improvements of EGFR-TKI monotherapy achieved were relatively modest. This retrospective study explores the possible benefit of chemotherapy combined with the first-generation TKI as neoadjuvant therapy for this group of patients, which has not been reported in the literature. However, compared with first-generation EGFR-TKI monotherapy, no significant improvements in short-term efficacy were found with combination therapy as neoadjuvant treatment. The ORR did not differ substantially, with 50.0% in the combination group and 40.5% in the monotherapy group, respectively (*P* = .495). No significant difference was found in DFS between the 2 groups (*P* = .832); however, the DFS data were still immature (only 40% relapsed in the combination group). While the combination group demonstrated a modest improvement in OS (83.81 vs 60.02 months), there was no significant difference (*P* = .809). Besides, the data from the combination group were still immature, with only 22.2% (4/18) of patients reaching the endpoint at the last follow-up.

Previous neoadjuvant studies conducted with first-generation EGFR-TKI in patients with stage III-N2 EGFR-mutant NSCLC have shown unsatisfying ORR ranging from 40% to 55%. Phase II clinical studies of erlotinib and gefitinib reported ORRs of 41.1%,^14^ 54.1%,^8^ and 54.5%,^15^ respectively, in resectable EGFR-mutant NSCLC. In this study, the ORR of the monotherapy group was 40.5%, consistent with previous data (71% of patients in this study were IIIA and 29% were IIIB). The combination of chemotherapy with first-generation EGFR-TKI yielded an ORR exceeding 80% for advanced EGFR-mutated NSCLC in the NEJ009 study. However, the ORR of combination therapy for EGFR-mutated III-N2 NSCLC was 50%, significantly lower than reported in NEJ009. The duration of treatment in the combination group (3 months), was not significantly shorter compared to other studies, suggesting that the treatment duration may not be the reason for the low ORR observed in our study. Several reasons may account for the lower ORR. Firstly, the biological behavior of advanced-stage NSCLC is generally more aggressive than that of middle-stage NSCLC, which could lead to increased TKI sensitivity. Secondly, the high proportion of patients with the exon 21 L858R mutation in the combination group (11/15) may have contributed to the lower ORR. It has been established that patients with advanced NSCLC with the exon 21 L858R mutation generally do not derive as much benefit from TKI monotherapy compared to patients with exon 19 deletion.^16^ While TKI combined with chemotherapy has been suggested to improve PFS and ORR compared with TKI monotherapy,^17^ this combination is considered a treatment option for advanced EGFR-mutated NSCLC in the real world. However, previous studies showed that the ORR of combination therapy in patients with exon 21 L858 mutation was lower than in patients with exon 19 deletion.^11^ In addition, one patient with PD in the combination group of our study had squamous cell carcinoma with EGFR mutation. It is known that therapeutic responses in patients with squamous cell carcinoma are generally suboptimal. However, with the application of the third-generation TKI in neoadjuvant treatment, a phase II study demonstrated that osimertinib achieved a higher ORR rate (71.1%),^18^ which was superior to results reported for first-generation EGFR TKI in neoadjuvant therapy studies. Chemotherapy combined with osimertinib also achieved a surprising 90.9% ORR rate^19^ in patients with advanced NSCLC with EGFR mutations. Accordingly, third-generation TKI monotherapy or combined with chemotherapy represents a promising option for neoadjuvant therapy in the future.

Previous neoadjuvant studies with first-generation TKIs have shown a low proportion of PCR, with no PCR observed in neoadjuvant phase II studies of erlotinib.^8,14^ In neoadjuvant phase II studies of gefitinib,^15^ the PCR rate was 12.1% (4/33), while in a neoadjuvant study of osimertinib,^18^ only 1 (3.6%) patient achieved PCR despite a significantly increased ORR. In this study, PCR was found in 3 (7.89%) patients in the monotherapy group, implying that even first-generation EGFR-TKIs may still have a good therapeutic effect in III-stage patients with EGFR mutations as neoadjuvant therapy. Identifying and screening biomarkers in this patient population could be important to help select individuals who would benefit from neoadjuvant targeted therapy. The prevalence rates of MPR in the combination and monotherapy groups in this study were 20.0% and 10.5%, respectively, similar to the MPR rates documented in previous TKI neoadjuvant research. However, the combination did not significantly improve the MPR (*P* = .359). Among the patients who achieved MPR in both groups, only one patient relapsed 6 months after surgery, consistent with the view that postoperative MPR is typically associated with improved OS.

The primary objective of neoadjuvant targeted therapy is to achieve downstaging, specifically by reducing the T stage and decreasing N2 disease to improve R0 resection rates. The R0 resection rates in both groups in this study were acceptable. Only one patient in each group underwent R1 resection, and the rest achieved R0 resection. The R0 resection rates were 93.3% versus 97.3% in the combination and monotherapy groups, respectively, indicating successful surgical outcomes. Previous studies have shown that patients with N2 downstaging after neoadjuvant chemotherapy can achieve a more favorable survival time.^20,21^ In this study, we sought to assess the N2 downstaging rate of patients with III-N2 NSCLC with EGFR mutation who received chemotherapy combined with the first-generation TKI. Although the N2 downstaging rate was higher in the combination group than in the monotherapy group (53.3% and 42.1%), the difference was not significant, implying that the N2 downstaging effect of chemotherapy combined with the first-generation EGFR-TKI therapy may not have been as effective as anticipated.

The emergence of neoadjuvant immunotherapy has led to significantly higher PCR and EFS rates in patients with stage III NSCLC with negative driver genes,^3^ which prompts an interesting question: can immunotherapy combined with chemotherapy be used as neoadjuvant therapy in patients with stage III NSCLC with EGFR mutations? A retrospective research^22^ found that patients with EGFR-mutated NSCLC of stage II-III who received immunotherapy combined with chemotherapy as neoadjuvant therapy could achieve an ORR of 63.2%, with an MPR of 42.1% and a PCR of 10.5%. Notably, the MPR and PCR rates observed in this study were higher than those reported in previous neoadjuvant-targeted therapy studies. Determining which subset of patients with EGFR-mutated would benefit the most from this approach warrants further investigation.

In previous studies, the proportion of patients who did not undergo surgery after neoadjuvant targeted therapy ranged from 16% to 26%,^8,14,18^ while this proportion of patients in our study was not high (16.6% in the combination group and 9.5% in the monotherapy group). In the monotherapy group, one patient with PD failed to undergo surgery. Additionally, among the 3 patients who declined surgery, 2 had SD and 1 had PR. Three patients in the combination therapy group who refused surgery had SD.

The safety profile observed in this study was generally consistent with known safety profiles of EGFR-TKI monotherapy or combined with chemotherapy.^8,14,18^ Grade 3 AEs were reported in 6 patients (rash and abnormal liver function in the monotherapy group; decreased WBC count and liver function in the combination group). While anorexia, nausea, fatigue, rash, decreased WBC count and decreased platelet count occurred in more than 30% of the patients in the combination group, most of these events were mild to moderate (grades 1-2) and resolved either with or without medication. No new AEs were identified.

However, there are some limitations to this study. First, this is a retrospective analysis, and selection bias was inevitable. Secondly, the sample size of the combination group was small. Thirdly, the follow-up duration in the study was relatively short, and approximately 60% of patients in the combination group had not yet experienced disease recurrence at the time of analysis. At last, it is essential to acknowledge that the collection of patient materials solely relied on retrospective data based on medical history descriptions, which may introduce subjective bias when assessing specific CTCAE grades.

Future studies should address important issues, including identifying patients most likely to achieve PCR from neoadjuvant targeted therapy and whether there are any related biomarkers. Other neoadjuvant strategies, such as chemotherapy combined with the third-generation EGFR-TKI or chemotherapy combined with immunotherapy in patients with EGFR mutations as neoadjuvant, may be worthy of further exploration.

## Conclusion

We found that chemotherapy combined with first-generation EGFR-TKI could be used as neoadjuvant therapy for EGFR-mutated III-N2 NSCLC with acceptable toxicity. However, regarding indicators for short-term efficacy, such as ORR, MPR, and PCR, chemotherapy combined with the first-generation EGFR-TKI therapy was not significantly superior to first-generation EGFR-TKI monotherapy. To ascertain the potential benefits of combination therapy as a neoadjuvant treatment for patients with stage III-N2 EGFR-mutant NSCLC, it may be necessary to conduct a long-term follow-up study to evaluate its impact on disease-free survival and overall survival.

## Supplementary Material

oyae052_suppl_Supplementary_Tables_S1-S5

## Data Availability

The data that support the findings of this study are available from the corresponding author upon reasonable request.
