# Supplementary material for: Combination of EGFR-TKI and Chemotherapy Versus EGFR-TKI Monotherapy as Neoadjuvant Treatment of Stage III-N2 EGFR-Mutant Non-Small Cell Lung Cancer
Source: Oncologist. 2024 Mar 25;29(7):e932–40. doi: 10.1093/oncolo/oyae052 (PMC11224993; doi:10.1093/oncolo/oyae052)
Supplement: oyae052_suppl_Supplementary_Tables_S1-S5 [file oyae052_suppl_supplementary_tables_s1-s5.docx]

**Supplementary tables**

Table S1. Targeted therapeutic drugs received as neoadjuvant treatment

| EGFR-TKIs | EGFR-TKI combined with chemotherapy(n=18) (%) | EGFR-TKI monotherapy (n=42) (%) |
| --- | --- | --- |
| Erlotinib | 6(33.3) | 13(31.0) |
| Gefitinib | 12(66.7) | 29(69.0) |

Table S2. Duration of treatment received before surgery

| Duration of treatment, months | EGFR-TKI monotherapy(n=38) (%) | | EGFR-TKI combined with chemotherapy(n=15) (%) | | |
| --- | --- | --- | --- | --- | --- |
| 1 | 4(10.5) |  | | 2(13.3) | |
| 2 | 28(73.7) |  | | 10(66.7) | |
| 3 | 6(15.8) |  | | 3(20.0) |  |

Table S3. Adjuvant therapy received after surgery

|  | Adjuvant therapy | EGFR-TKI monotherapy(n=38) (%) |  | EGFR-TKI combined with chemotherapy(n=15) (%) | *P* value |
| --- | --- | --- | --- | --- | --- |
| Adjuvant  EGFR-TKI with  or without chemotherapy | EGFR-TKI monotherapy | 11(28.9) |  | 6(40.0) | 0.172 |
|  | EGFR-TKI followed radiotherapy | 0(0.0) |  | 1(6.7) |  |
|  | EGFR-TKI combined with chemotherapy | 1(2.6) |  | 1(6.7) |  |
| Other treatments | Platinum-based chemotherapy | 11(28.9) |  | 4(26.7) |  |
|  | Chemotherapy combined with radiotherapy | 4(10.5) |  | 1(6.7) |  |
|  | Without adjuvant therapy | 11(28.9) |  | 2(13.3) |  |

Table S4. Characteristics of patients who received adjuvant TKI therapy after surgery

| group | Patients | Gender | Age | TNM | EGFR mutations | TKI drug | Duration of treatment (months) |
| --- | --- | --- | --- | --- | --- | --- | --- |
| EGFR-TKI monotherapy | 1 | female | 56 | T3N2M0 | 19deletion | Osimertinib | 18 |
|  | 2 | female | 48 | T1cN2M0 | 19deletion | Osimertinib | 30 |
|  | 3 | male | 58 | T2N2M0 | 19deletion | Gefitinib | 6 |
|  | 4 | male | 70 | T3N2M0 | 19deletion | Gefitinib+ chemotherapy | 9 |
|  | 5 | male | 60 | T2N2M0 | 21 L858R | Osimertinib | 14 |
|  | 6 | male | 59 | T2N2M0 | 21 L858R | Erlotinib | 11 |
|  | 7 | male | 67 | T1N2M0 | 21 L858R | Erlotinib | 21 |
|  | 8 | male | 60 | T1N2M0 | 21 L858R | Erlotinib | 22 |
|  | 9 | male | 63 | T1N2M0 | 19deletion | Osimertinib | 30 |
|  | 10 | female | 59 | T2N2M0 | 19deletion | Erlotinib | 36 |
|  | 11 | female | 60 | T2N2M0 | 19deletion | Gefitinib | 8 |
|  | 12 | female | 75 | T3N2M0 | 19deletion | Gefitinib | 30 |
| EGFR-TKI combined with chemotherapy | 13 | male | 51 | T2N2M0 | 21 L858R | Osimertinib | 24 |
|  | 14 | female | 73 | T2N2M0 | 21 L858R | Gefitinib | 22 |
|  | 15 | female | 59 | T3N1M0 | 21 L858R | Gefitinib | 24 |
|  | 16 | female | 68 | T4N2M0 | 19deletion | Gefitinib+ chemotherapy | 8 |
|  | 17 | female | 48 | T2N2M0 | 19deletion | Osimertinib | 12 |
|  | 18 | male | 68 | T1N2M0 | 21 L858R | Osimertinib | 12 |
|  | 19 | male | 44 | T4N2M0 | 19deletion | Erlotinib+ radiotherapy | 36 |
|  | 20 | female | 55 | T1N2M0 | 21 L858R | Gefitinib | 24 |

Table S5. Adverse events during neoadjuvant therapy

|  | Adverse events | CTCAE grade 1, n(%) | CTCAE grade 2, n(%) | CTCAE grade 3, n(%) | | CTCAE grade 4, n(%) |
| --- | --- | --- | --- | --- | --- | --- |
| EGFR-TKI  (n=42) | Rash | 10(23.8) | 5(11.9) | 1(2.4) | / | |
|  | Diarrhea | 11(26.2) | / | / | / | |
|  | Paronychia | 5(11.9) | / | / | / | |
|  | Anorexia | 18(42.9) | / | / | / | |
|  | Nausea | / | / | / | / | |
|  | Fatigue | 4(9.5) | / | / | / | |
|  | Decreased WBC count | 1(2.4) | / | / | / | |
|  | Decreased Platelet count | / | / | / | / | |
|  | Abnormal liver function | 5(11.9) | / | 2（4.8） | / | |
| EGFR-TKI  combined with  chemotherapy  (n=18) | Rash | 6(33.3) | 1(5.5) | / | / | |
|  | Diarrhea | 5(27.8) | / | / | / | |
|  | Paronychia | 5(27.7) | / | / | / | |
|  | Anorexia | 9(50.0) | 3(16.6) | / | / | |
|  | Nausea | 10(55.5) | 1(5.5) | / | / | |
|  | Fatigue | 8(44.4) | / | / | / | |
|  | Decreased WBC count | 3(16.7) | 2(11.1) | 2(11.1) | / | |
|  | Decreased Platelet count | 4(22.2) | 3(16.7) | / | / | |
|  | Abnormal liver function | 4(22.2) | / | 1(5.5) | / | |
